# Scratching the Surface: Lipidomic Profiling of the Stratum Corneum in the Search for Pruritogens in Cholestatic Liver Diseases

**DOI:** 10.3390/jpm16070391

**Published:** 2026-07-22

**Authors:** Rebecca L. Beres, Kenneth D. R. Setchell, Marialena Mouzaki, Xueheng Zhao

**Affiliations:** 1 Division of Pathology and Laboratory Medicine, Cincinnati Children’s Medical Center, Cincinnati, OH 45229, USA; rebecca.beres@cchmc.org (R.L.B.);; 2 Department of Pediatrics, University of Cincinnati College of Medicine, Cincinnati, OH 45267, USA; 3 Division of Gastroenterology and Nutrition, Cincinnati Children’s Medical Center, Cincinnati, OH 45299, USA

**Keywords:** cholestatic pruritus, lipidomics, stratum corneum, biomarker, tape stripping

## Abstract

Pruritus is a debilitating symptom frequently affecting patients with cholestatic liver disease, often resistant to conventional antipruritic therapies. The pathogenesis of cholestatic pruritus (CP) is multifactorial, implicating not only bile acids but also a complex array of other potential pruritogenic mediators and neural signaling pathways. Identification of the exact pruritogen has been elusive, and gaps remain in understanding the pathogenesis of pruritus, so developing targeted treatments is critical. The stratum corneum (SC), the outermost lipid-rich layer of the skin, may act as a reservoir for circulating pruritogens, offering a novel window to explore the pathogenesis of CP. Recent advancements in lipidomics and non-invasive tape stripping have enabled detailed profiling of SC lipid alterations in disease states. In this review, we synthesize the current understanding of CP and its candidate pruritogens and describe state-of-the-art approaches for SC lipid analysis, combining tape stripping to sample the skin surface with mass spectrometry-based lipidomics. Finally, we summarize cutaneous molecular findings and discuss how these techniques are facilitating biomarker discovery and informing therapeutic development. This review discusses the paradigm shift from a single-molecule perspective to a more integrated view of CP as a product of complex mediator interactions and highlights the potential of SC profiling to uncover novel targets for intervention strategies.

## 1. Introduction

Pruritus is one of the most enigmatic symptoms of cholestatic liver diseases. Patients with different cholestatic liver diseases frequently experience intense and sometimes debilitating itch, often becoming worse at night [1,2]. Over time, this not only causes sleep deprivation and depression but also becomes a main contributor to poor quality of life. The intense itch often elicits repetitive scratching that damages the skin barrier, potentially releasing more pruritogens and creating a self-perpetuating vicious cycle. Unlike many other forms of itch, traditional and historical treatments are usually ineffective for the relief of CP [3]. For decades, and despite many proposed candidates, the exact pruritogen has remained unidentifiable, representing a major gap in understanding CP therapeutic development and hampering potential therapies.

Pruritogens can be defined as endogenous or exogenous mediators capable of initiating or amplifying itch signaling through either direct activation of sensory neurons or indirect activation of inflammatory and neuroimmune pathways [3,4]. These mediators can be categorized into various classes, such as lipid mediators, peptides, steroid metabolites, neurotransmitters and amines, cytokines, and enzyme-regulated signaling systems [4,5,6,7]. While many cholestatic pruritogens are small molecules, evidence suggests that itch generation could result from interactions among multiple mediators, rather than a single factor [3,4]. Understanding the contributions of these pruritogen classes is essential to target therapies, and it remains a major challenge in the search for the pathogenesis of cholestatic pruritus.

The search for cholestatic pruritogens has been a long scientific journey. Cholestasis is defined as impaired bile formation and/or bile flow and, beyond maldigestion, potentially may lead to clinical symptoms of pruritus and fatigue, but not in all patients with cholestasis. Bile acids were first proposed as the potential pruritogen more than 50 years ago, based on the finding of elevated serum bile acids in pruritic patients with severe cholestasis, as well as early observations that application of bile and bile acids to dekeratinized skin and blister bases induced itch [8]. There is a poor correlation, however, between serum bile acid levels and itch severity [4,9]. Treatments such as cholestyramine or ileal bile acid transporter (iBAT) inhibitors may reduce pruritus in some patients, concomitant with reduced serum bile acid concentrations [10,11,12,13]; however, these findings alone do not definitively establish bile acids as the main pruritogen. Moreover, a lowering of serum bile acids is consistent with relief of cholestasis and improvement in bile flow, which, in turn, should relieve the itch. Other potential pruritogens, including lysophosphatidic acid (LPA), autotaxin (ATX), endogenous opioids, and steroids, have also been proposed as candidate molecules [4,6,7], but the associations are seemingly circumstantial.

**Figure 1:**
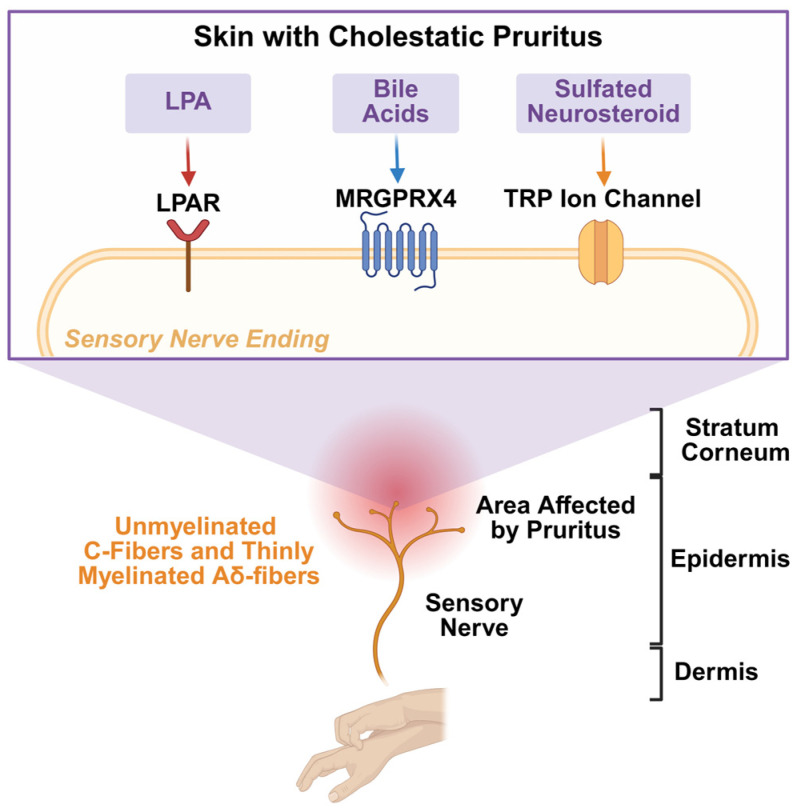
Illustration of potential mechanisms of cholestatic pruritus (CP) in the skin (created with BioRender.com). Pruritogens, such as lysophosphatidic acid (LPA), bile acids, and sulfated neurosteroids, may activate sensory nerve fibers that innervate the skin. Itch is subsequently transmitted primarily through Aδ and C fibers to the spinal cord. This figure highlights selected cholestatic pruritogens and does not depict all potential pruritogenic mediators, including cytokines and other neuroimmune signaling molecules. MRGPRX4: Mas-related G protein-coupled receptor X4; LPAR: lysophosphatidic acid (LPA) receptors; TRP: transient receptor potential ion channels.

Because of the importance of identifying candidate pruritogens, a deeper understanding of itch signal processes and how they arise is crucial. In cholestatic pruritus, sensory input begins in the skin, where signals are relayed via peripheral pathways to the central nervous system [2]. As shown in Figure 1**,** various mediator classes could influence this process, mainly by altering the responsiveness of cutaneous sensory pathways [14]. Although the precise mechanism remains uncertain, evidence suggests that cholestasis can modify the chemical environment of the skin and subsequently increase exposure to circulating as well as locally generated metabolites contributing to itch [4,5,15,16,17]. Notably, elevations in pregnanolone sulfates, for example, have been reported to be linked to the pruritus associated with intrahepatic cholestasis of pregnancy (ICP) [18,19,20], which interestingly resolves after delivery of the newborn and reoccurs with subsequent pregnancies.

Beyond signaling, the skin itself may serve as a metabolic site of pruritogen production or modification. Importantly, mounting evidence positions the skin not merely as a passive barrier but as an organ that actively communicates with the central nervous system through local and systemic factors, including lipids, hormones, and neurotransmitters [21,22,23]. This complex itch mechanism implies that optimal management requires therapies that are specifically tailored directly or indirectly to the pruritogenic pathways. Emerging therapies like iBAT inhibitors and peroxisome proliferator-activated receptor (PPAR) agonists have shown promise [24,25,26], although these are not effective in every patient for unknown reasons. Their mechanism of action and efficacy, however, require further elucidation, and the need to identify definitive pruritogenic drivers remains to be defined.

This review aims to provide a narrative of how the combined use of tape stripping and mass spectrometry-based analytical techniques could facilitate pruritogen discovery and highlight a paradigm shift from a “single-molecule” theory to a complex interplay of several potential mediators. We also outline how these approaches are paving the way for biomarker discovery in liver diseases in general.

## 2. Stratum Corneum: More than a Barrier?

**Figure 2:**
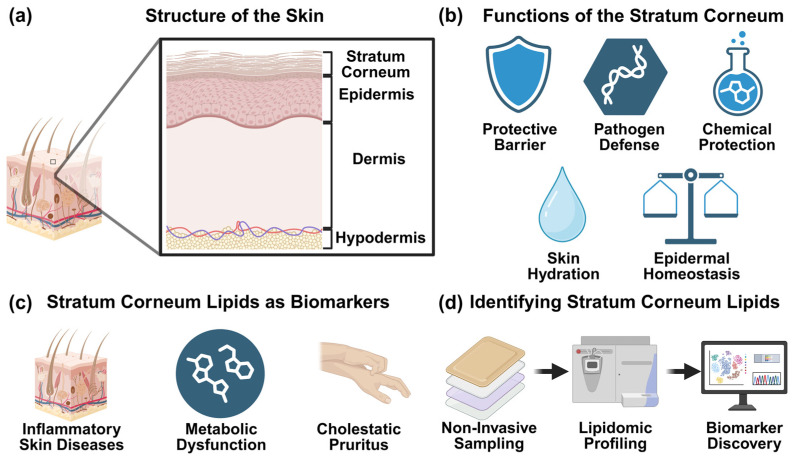
Overview of the structure, function, and clinical relevance of the stratum corneum (SC) (created with Biorender.com). (**a**) Structure of the skin showing that the SC is the outermost layer above the epidermis, dermis, and hypodermis. (**b**) Key functions of the SC. (**c**) Schematic illustrating how SC lipids can be used as biomarkers in inflammatory skin diseases, including pruritus and other systemic conditions. (**d**) Workflow for SC lipidomic analyses.

The stratum corneum (SC) is the outermost layer of the epidermis and serves as the body’s primary interface with the external environment. It typically ranges from 10 to 20 µm in thickness [27,28] and consists of terminally differentiated keratinocytes, found within a lipid-rich extracellular matrix [29,30]. Because of its unique organization, the SC functions as an effective barrier in protecting against pathogen invasion and chemical exposures [31] and limiting damage from UV radiation [32]. It is also critical to maintain skin moisture by limiting transepidermal water loss. An overview of SC structure, function, and relevance to lipidomic biomarker discovery is shown in Figure 2.

Lipids comprise roughly 10–20% of the SC by weight, with ceramides (Cer), cholesterol (Chol), and free fatty acids accounting for the main lipid classes [29]. These lipids are arranged in lamellar structures, crucial for maintaining skin functions, including barrier integrity and hydration [33,34]. Ceramides are essential, with important functions in regulating permeability and organization of the SC [35,36]. Alterations to SC lipids have been linked to numerous dermatological and inflammatory skin conditions, including atopic dermatitis, xerosis, psoriasis [22,37], metabolic dysfunction [38], and pruritus [21].

Since the skin, and particularly the SC, can act as a reservoir that accumulates many exogenous and endogenous substances, detailed analysis of its composition may provide insights into metabolic changes within the body, beyond those identified from blood sampling or urine analysis. The composition of the SC will reflect physiological processes as well as responses to environmental stimuli, making it a sensitive indicator of skin health and barrier integrity [39,40]. Moreover, analysis of SC samples would represent the cumulative effects of metabolites over many days, rather than the transient fluctuations seen in blood or urine, which could enhance the identification of disease-specific biomarkers, such as potential pruritogens [29,41].

Historically, the possible role of bile acids in the skin was explored using vacuum blistering techniques to separate the epidermis from the dermis, with sampling of the interstitial fluid generated by the blister [42,43]. Alternatively, punch skin biopsies were obtained [44]. In both approaches, no definitive evidence was obtained to implicate bile acids as the pruritogen. Additionally, these approaches are relatively invasive, limiting the ability for repeated or routine sampling. In contrast, the SC can be sampled using non-invasive techniques such as tape stripping, enabling repeated measurements with minimal burden on study participants, as well as providing an effective approach for investigating the lipidome of the skin [45,46].

**Figure 3:**
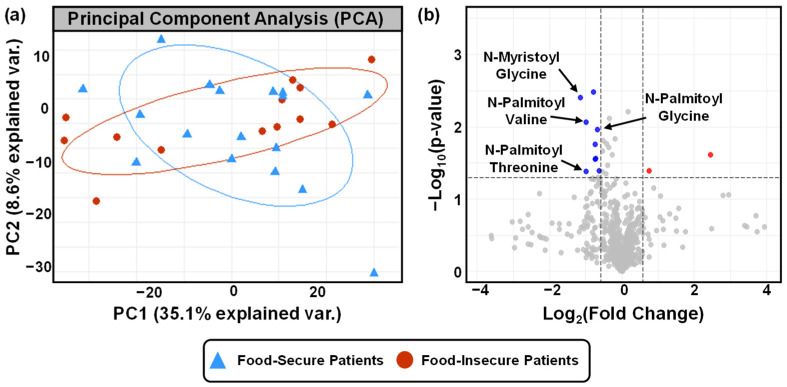
Untargeted lipidomics analysis of the stratum corneum between food-insecure and food-secure patient groups. (**a**) Principal component analysis (PCA) score plot illustrating the lipidomic profiles of stratum corneum samples. Each point represents an individual sample, with the variance explained by each component shown on the axes. (**b**) Volcano plot displaying differential lipid features between food-secure and food-insecure patient groups. Red dots indicate significantly upregulated lipids (log_2_ fold change >1, *p* < 0.05), blue dots indicate significantly downregulated lipids (log_2_ fold change <−1, *p* < 0.05), and gray dots indicate lipids that did not meet significance criteria. The vertical dashed lines denote the log_2_ fold change thresholds (±1), and the horizontal dashed line indicates the significant threshold (*p* = 0.05; −log_10_(*p*) = 1.30). No correction for multiple testing was performed in this analysis. Considering the high dimensionality of the dataset and the limited sample size, the findings should be regarded as exploratory and intended primarily to generate hypotheses for future validation studies. Institutional Review Board (IRB) approval with IRB ID 2019-1178 (Cincinnati Children’s Hospital Medical Center) and 2000030240 (Yale New Haven Children’s Hospital).

Recent advances in lipidomic methodologies further enhance the ability to profile a vast number of lipid species [46,47,48]. Previous studies have showcased the relationship between biologically meaningful changes in SC lipid composition and barrier dysfunction [22], inflammatory signaling [23], and accumulation of pruritogenic mediators [21]. More recently, SC lipidomes were analyzed between food-insecure and food-secure pediatric patients, showing how nutritional status may influence skin barrier lipid composition and function [49]. In this study, untargeted lipidomics analysis was conducted on a high-resolution Q Exactive^TM^ plus Orbitrap mass spectrometer with an ultra-high-performance liquid chromatography (UHPLC) system. A reverse-phase C18 UPLC column was used to separate metabolites, and data were acquired using full MS scan and collision-induced dissociation-based data dependent on MS/MS. Lipid annotation was conducted by searching against the in-house lipidomics database using retention time (RT), accurate mass and fragmentation ion pattern. To account for the varied protein levels in skin tapes, median fold change (MFC) was applied to normalize the peak intensities. Skin metabolites, including both polar and nonpolar lipid species, were extracted from the tapes using an optimized method with internal standards. Multivariate and univariate analyses of lipidomics data from SC samples collected from food-secure and food-insecure patients identified differences in lipid profiles and potential lipid biomarkers [49], as illustrated by principal component analysis (PCA) and volcano plot analysis (Figure 3). While this exploratory study is limited by a small sample size, the results suggest that the SC may serve as a dynamic and clinically informative resource for studying and searching for biomarkers.

## 3. Pruritogens in Cholestatic Liver Diseases

In cholestatic liver diseases, the intensity and frequency of pruritus are not directly associated with conventional biochemical liver function tests, such as serum bilirubin, alanine aminotransferase (ALT), and alkaline phosphatase (ALP) [9,50]. Elevated liver function tests are frequently observed in patients with cholestasis who do not present with pruritus, raising the question of what underlies the etiology of cholestatic pruritus. These inconsistencies have suggested that additional biochemical mediators drive pruritus.

Relief of pruritus following the administration of cholestyramine, an anion exchange resin, as shown in ref. [13], caused a decrease in pruritus, with significant reductions in serum bile acid concentrations, which lent further support to the idea that bile acids caused pruritus. More recently, iBAT inhibitors have been approved for the treatment of pruritus, with relief responses associated only with significant decreases in serum bile acid concentrations, whereas treatment failures were associated with a failure to achieve a significant reduction in this endpoint [10,11,12]. Cholestyramine, on the other hand, also relieves pruritus-associated uremia, a condition in which serum bile acids are not elevated [51,52]. In our opinion, these findings are more consistent with an improvement in bile flow than with bile acids themselves acting as the primary pruritogen [13]. Improved understanding of bile acid-responsive receptors, including MRGPRX4, FXR, and PPAR, has accelerated the development of targeted therapies for CP [53,54]. Agonists of PPAR [55,56] and FXR [57,58], as well as modulators of MRGPRX4 signaling [59,60], have demonstrated therapeutic potential in preclinical studies. These agents not only suppress bile acid-induced pruritic behaviors but also improve biochemical markers of cholestatic disease activity. Notably, clinically viable agonists and antagonists of MRGPRX4, a bile acid-sensing G protein-coupled receptor implicated in CP, have been studied and identified for CP treatment recently through high-throughput screening and structure-activation-guided modeling approaches [53,59]. Collectively, these findings highlight bile acid signaling pathways as promising therapeutic targets. Nevertheless, further studies are needed to elucidate how bile acids and related metabolites interact with sensory neurons and immune cells and how pruritus signaling in CP is evoked and sustained in different cutaneous compartments.

Beyond bile acids, various mediators have been identified and linked to the development of cholestatic pruritus. Previous studies have identified other mediators of pruritus, including endogenous opioid peptides, which act to modulate itch perception [7,61]. Increased activity of µ-opioid receptors has been linked to enhanced pruritic responses, whereas opioid antagonists have been used in clinical settings to help alleviate cholestatic itch [62]. Likewise, small molecules, such as histamine and serotonin, can also stimulate peripheral pruriceptive neurons, but their contribution to CP appears less significant when compared to dermatologic or allergic itch [3,7]. More recently, lysophospholipids, including lysophosphatidic acid (LPA), have emerged as potential mediators of pruritus [4,9], and the detailed mechanisms and clinical relevance are discussed further in this review. Many of these candidate pruritogens are lipid or lipid-like molecules, likely derived from altered systemic lipid metabolism in the presence of cholestasis. It is therefore reasonable to hypothesize, as we do, that the SC may serve as a “sink” or “reservoir” for circulating lipid pruritogens, although direct experimental evidence in CP is still limited.

## 4. SC Lipids and Association with Liver Diseases

Lipids play crucial roles in liver physiology, including maintaining hepatic structure [63], regulating metabolic function, and mediating intracellular signaling pathways [64]. The liver is critical to maintaining lipid homeostasis in the body, with roles in lipid uptake, synthesis, storage, and export. When these processes are disrupted, changes in lipid composition occur, often leading to hepatic dysfunction [65,66,67]. Additionally, disease progression does not depend on lipid burden alone but also on the accumulation of lipid species known to promote lipotoxicity and disrupt cell signaling [68,69]. Inflammatory responses, metabolic stress, and cell survival can be influenced by lipid species, contributing to the probability of liver injury.

Among these additional lipid-related mediators, lysophosphatidic acid (LPA), a bioactive lipid generated by the enzyme autotaxin (ATX), has emerged as a potential candidate for CP [4]. Previous studies have indicated that elevated LPA levels are observed in severe pruritus, with other experimental mouse models suggesting that the administration of LPA induces scratching behavior [15,70]. While bile acids and LPA are not directly connected through a shared metabolic pathway, evidence from cholestatic liver disease suggests that bile acid accumulation and dysregulated signaling can create a pro-inflammatory and fibrotic hepatic environment, which can potentially enhance ATX expression and LPA production via changes in phospholipid metabolism [71,72]. These findings, therefore, support the theory that bile acids may act upstream by shaping the cholestatic metabolic environment, while LPA functions downstream as an effector for lipid-mediated pruritogenic signaling.

Beyond bile acids and LPA, cholestatic liver disease is often characterized by the widespread remodeling of hepatic lipid metabolism, which contributes to hepatocellular injury, inflammation, and fibrosis. Altered phospholipid homeostasis is often linked to cholestasis and hepatobiliary injury, leading to increased lysophospholipid species and compromised membrane integrity [73,74]. Additionally, sphingolipid metabolism is affected, as the accumulation of ceramides and sphingosine can often induce hepatocyte apoptosis and mitochondrial dysfunction [64,75]. Likewise, fatty acid metabolism can also be affected, since bile acid dysregulation can promote the accumulation of saturated free fatty acids, lipid droplet formation, and mitochondrial stress [69,75,76]. Cholesterol and cholesterol ester homeostasis are similarly affected, with retention of cholesterol and lipoprotein-X formation contributing to cytotoxicity [75,77]. Lipid alterations can be found in a cell-specific manner, such as changes in lipid composition in hepatocytes, hepatic stellate cells, and other subcellular organelles that may differentially influence inflammation, fibrogenesis, and cellular stress responses [78,79], contributing to the systemic lipid dysregulation commonly observed in cholestasis.

The pathophysiology of cholestatic pruritus involves multiple factors, including biochemical mediators, signaling pathways, and other organ-specific interactions. Previous studies have suggested that altered spinal cord processing, changes in neurotransmitter release, and modulation of central opioid pathways can contribute to CP [3,5]. This complex interplay between peripheral and central triggers likely explains why single-target therapies frequently provide incomplete relief. Understanding these mechanisms is vital for the development of effective therapies, particularly those targeting lipid mediators and their downstream signaling pathways, which are further discussed in this manuscript. Additionally, cholestatic liver disease is often characterized by lipid dysregulation, including alterations in lipid classes such as bile acid-derived lipids, phospholipids, sphingolipids, and cholesterol esters [64,69,75]. Among these, bile acids function as signaling molecules that contribute to metabolic, inflammatory, and fibrotic pathways via receptors such as the farnesoid X receptor (FXR) and Takeda G protein-coupled receptor 5 (TGR5) [73,74]. In cholestasis, dysregulated bile acid homeostasis and disrupted signaling may contribute to broader alterations in hepatic lipid metabolism, promoting inflammation, fibrogenesis, and hepatocellular injury and disease [6,8].

Recent advances in lipidomics and metabolomics have enabled profiling of disease-specific lipid alterations, offering new opportunities to discover mechanisms underlying hepatic disorders [40,45,80]. This emerging evidence indicates that lipid dysregulation in cholestatic disease extends beyond the liver. The potential of tissue-specific lipidomic profiling is highlighted by these findings, with the analysis of the skin and SC being used to better understand pruritus pathogenesis, as well as to identify novel biomarkers and potential therapeutic targets.

## 5. Technological Advancements to Enable SC Lipidomic Biomarker Discovery

Over the past decade, advances in technology have enhanced the ability to extract, separate, identify, and quantify thousands of lipids with high sensitivity, spatial resolution, and confidence. Combined with a tape stripping technique, this technological advance provides specialized analytical strategies for evaluating the SC metabolome to preserve not only vertical resolution but also to achieve sufficient sensitivity and structural specificity [47,48,80].

**Figure 4:**
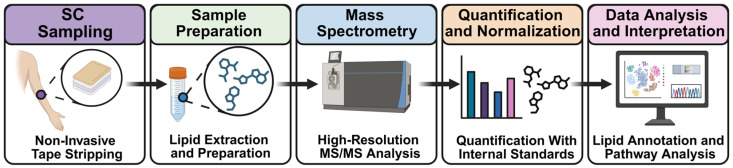
Workflow for SC lipidomic analysis (created with Biorender.com). Sampling of the SC is done using non-invasive tape stripping, followed by sample preparation using extraction methods tailored to the objective of the study. Samples are subsequently analyzed by high-resolution mass spectrometry (HRMS and MS/MS) to achieve comprehensive lipidomic profiling. Quantification is then performed using strategies tailored to the study objective, and the data is subjected to further analysis.

Non-invasive tape stripping removes sequential layers of the SC that will subsequently permit profiling of the cutaneous biochemical environment where pruritus is generated. The standardization of tape stripping protocols [81], the number of strips [47], and sequential collection [48] has improved reproducibility and further enabled in-depth resolved profiling, as shown in Figure 4, which also summarizes the overall workflow for SC lipidomic analyses from sampling to data interpretation.

In addition to optimized sampling strategies, extraction protocols have evolved to address the unique challenges of SC lipidomics analysis. Unique challenges arise when extracting lipids from skin tape samples, as the polymers within the adhesive tapes interact with certain concentrations of chloroform, forming a gel-like matrix that complicates lipid recovery [48]. Furthermore, solvent systems must efficiently solubilize both polar and nonpolar lipids. To address these challenges, both biphasic and monophasic extraction methods have been adapted specifically for skin tape samples, ensuring efficient separation of polar and nonpolar lipids directly from the tape [47,82]. Additional measures have been taken to achieve the maximum possible lipid recoveries, ranging from temperature-controlled incubation to extended sonication steps and stepwise extraction protocols [47,82]. Moreover, using isotopically labeled lipid standards that span multiple lipid classes and chain lengths facilitates accurate quantification and assessment of extraction efficiency [82]. Collectively, these extraction enhancements provide a robust framework for downstream chromatographic separation, mass spectrometry, and ultimately depth-resolved lipidomic profiling of the SC.

Advances in liquid chromatography–tandem mass spectrometry (LC-MS/MS) have also been central to enabling comprehensive lipidomic profiling of the SC. Because of the molecular diversity of lipids found in the SC, it is necessary to perform LC-MS/MS separations to not only mitigate matrix effects and ion suppression but also to resolve isobaric and isomeric species [83,84]. Reverse-phase liquid chromatography is mostly used to separate lipids due to the ability to separate lipids based on fatty acyl chain lengths and degrees of unsaturation. Mass spectrometry instrumentation has also improved, with enhanced sensitivity, mass accuracy, and structural confidence through data-dependent and data-independent tandem MS acquisition strategies [85,86]. Additionally, advances in automated peak detection, spectral library matching, and lipid annotation software have boosted confidence in lipid identification [87,88].

Lipidomic workflows can generally be divided into targeted and untargeted strategies, each serving complementary roles depending on the study objectives. Untargeted lipidomics enables broad profiling of hundreds of lipid species and is particularly valuable for biomarker discovery and hypothesis-generating studies [83,85]. In contrast, targeted lipidomics focuses on predefined lipid classes or molecular species, offering improved sensitivity, selectivity, and quantitative accuracy, especially since metabolite concentrations are measured rather than fold changes. Given that many pruritogens and signaling lipids are present at low abundance, targeted approaches are well suited to validating biomarkers identified in untargeted analyses and to achieving more reliable quantification across samples.

Several additional analytical considerations are essential to ensure robustness and reproducibility in lipidomic studies. Batch effects arising from sample preparation, retention time drift, instrument variability, and long analytical worklists can introduce systemic variability that confounds biological interpretation. To address this, rigorous quality control (QC) strategies, such as pooled QC samples, randomized acquisition lists, and signal drift corrections, are increasingly incorporated into lipid workflows [85,86]. These practices support both data normalization and assessment of run quality. In addition, structural confirmation of lipid species remains a key challenge in lipidomics, especially for isomeric and isobaric species. High-confidence annotation typically requires integration of tandem mass spectrometry fragmentation patterns, retention time characteristics, and high-resolution accurate mass measurements, and these factors may benefit from orthogonal validation strategies where feasible [83,84,87,88].

**Figure 5:**
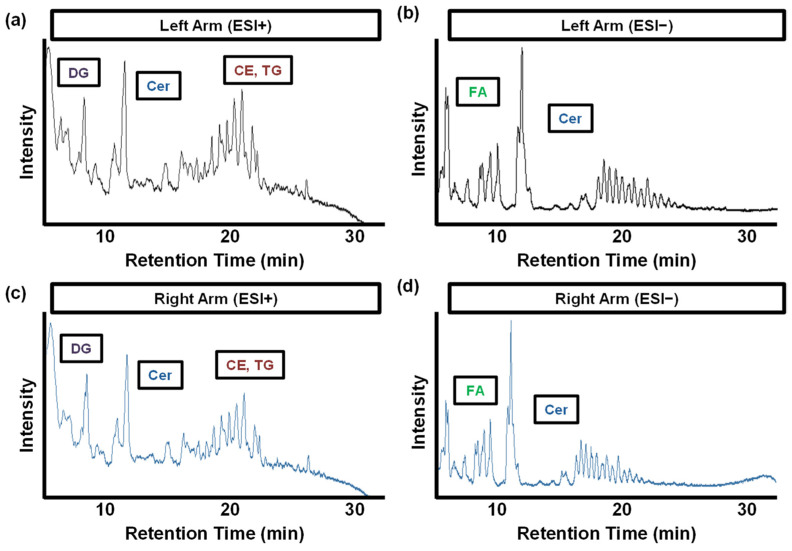
Typical total ion chromatograms (TICs) of stratum corneum lipid extracts obtained in positive and negative electrospray ionization modes from left and right arm samples of a healthy adult. (**a**) Left arm analyzed in positive electrospray ionization mode (ESI+), showcasing prominent lipid classes, including diacylglycerols (DGs), ceramides (Cers), and cholesteryl esters/triglycerides (CEs/TGs). (**b**) Left arm analyzed in negative electrospray ionization mode (ESI-), with dominant lipid classes being free fatty acids (FAs) and ceramides (Cers). (**c**) Right arm analyzed in ESI+, illustrating a similar lipid distribution to the left arm, with DG, Cer, and CE/TG species being detected. (**d**) Right arm analyzed in ESI-, with FA and Cer species having comparable retention times. Samples were collected with Institutional Review Board (IRB) approval, IRB ID 2024-0835 (Cincinnati Children’s Hospital Medical Center).

Together, these advances have supported the development of depth-resolved characterization of the SC lipidome. Representative total ion chromatograms (TICs) acquired in positive and negative ionization modes of SC skin tapes simultaneously obtained from the left and right arms of healthy participants in our labs are shown in Figure 5, showcasing the complexity and reproducibility of lipidomic detection across samples.

## 6. The Stratum Corneum–Itch Axis: Linking SC Lipid Defects to Cholestatic Pruritus

Consistent with their role as a biological interface, circulating lipids can integrate into and further influence the SC matrix. Systemic lipids may be transported by diffusion through dermal capillaries or secretion by sebaceous glands, potentially leading to partitioning into the extracellular lipid lamellae of the SC and influencing local lipid synthesis and metabolism [15,70,71]. Cholestatic lipid dysregulation could subsequently drive measurable changes in the cutaneous lipidome. For instance, lipidomic changes include shifts in ceramide composition, increased lysophospholipid species, or other bioactive lipids with potential roles in itch signaling [64,73,75]. With these structural changes, barrier integrity may be compromised, facilitating enhanced penetration and retention of circulating pruritogens within the epidermis.

Cholestatic pruritus involves multiple peripheral and central pathways. Pruritogens, whether endogenous or exogenous, presumably bind to their target receptors on cutaneous nerve fibers, triggering depolarization and signal propagation. To feel itch, sensation is first initiated in the skin and transmitted via specialized peripheral neurons, primarily unmyelinated C-fibers and thinly myelinated Aδ-fibers. Pruritogens activate peripheral nerve endings in the skin, transmitting signals through the dorsal root ganglia to the spinal cord [2]. Signal transduction at the skin relies heavily on two principal receptor families: G protein-coupled receptors (GPCRs) and transient receptor potential (TRP) channels. Human bile acid and bilirubin receptors, LPA receptors, and opioid receptors, members of the GPCR class, mediate itch signaling and potentiate itch after activation in cholestasis, either directly or indirectly. TRP channels, on the other hand, function downstream of GPCRs to convert chemical signals from pruritogens to neuronal excitation, subsequently propagating to the spinal cord and evoking itch perception [89]. As a result, pruritogens in the skin may arise by local production, transport from circulation, or release from infiltrating immune cells. Candidate pruritogens, including lysophospholipids, sulfated pregnanolones, bile acids and bilirubin conjugates, may play either a direct or indirect role [5]. Additionally, sulfated steroids modulate ion channels and neurotransmitter receptors in a non-genomic, membrane-delimited manner, likely contributing to CP. Sulfated steroids modulate ion channels and neurotransmitter receptors in a non-genomic, membrane-delimited manner, likely contributing to CP. Furthermore, itch pathogenesis is a neuroimmune process in which immune cells and sensory neurons communicate with cytokines, chemokines, and lipid mediators, further modulating pruriceptive signaling at peripheral, spinal, and central levels [5,16,90].

Beyond reflecting systemic alterations, lipid changes within the SC may also contribute directly to pruritus pathogenesis. In cholestatic conditions, pruritogenic lipids, such as lysophosphatidic acid and oxylipins, may accumulate within the SC, increasing their availability at the skin surface and within the epidermal environment, though this has yet to be directly demonstrated in CP [15]. Oxylipins, for example, are not limited to being byproducts of skin inflammation but are also active itch mediators that bridge the immune and nervous systems. Oxylipins, such as leukotriene B4 (LTB4) and prostaglandin E2 (PGE2), signal through GPCRs on sensory neurons to promote itch [91,92]. Because of this, the balance between pro-itch (arachidonic acid-derived) and anti-itch (eicosapentaenoic acid (EPA)/docosahexaenoic acid (DHA)-derived, pro-resolving) oxylipins likely contributes to the determination of itch susceptibility and severity. With this in mind, altered bile acid metabolism may also influence and interact with oxylipin pathways, affecting itch. Additionally, because SC turnover occurs over multiple days to weeks, lipid alterations can prolong exposure of cutaneous sensory nerve endings to itch-inducing signals. Since SC lipid remodeling can extend beyond lipid accumulation, sensory nerve endings located in the epidermis can express receptors that are commonly responsive to lipid mediators, including GPCRs and TRP channels [73]. Likewise, activation of these receptors by accumulated lipids could trigger neuronal depolarization and further transmission of itch signals to the central nervous system, raising the possibility that SC lipid alterations could influence pruriceptive signaling.

In summary, this supports using the SC as a relevant tissue for understanding systemic lipid dysregulation, as well as translating it into potential biochemical and sensory effects. Profiling SC lipid species may enable correlation of specific lipid signatures with itch severity scores, providing an objective biochemical readout to complement patient-reported outcomes. In addition, longitudinal monitoring of SC lipid profiles could help track changes in pruritogenic mediators in response to therapies, offering a potential tool for evaluating treatment efficacy and guiding personalized interventions. Identifying specific pruritogenic lipids and pathways would support the development of targeted therapies aimed at lipid signaling and pathways or at restoring balance in the skin.

## 7. Limitations and Challenges of SC Lipidomics in Biomarker Discovery of CP

Although there is growing utilization of SC lipidomics, several factors, including biological, technical, and clinical limitations, are currently present. These limitations are important to acknowledge as SC lipidomics is implemented for biomarker discovery and mechanistic insight and subsequently translated into clinical applications.

Biological variability remains one of the major challenges in SC lipidomic analyses. The lipidomic composition of the SC varies significantly, with aspects such as anatomical location, age, sex, ethnicity, environmental exposure, skin hydration, cosmetic product use, and underlying skin barrier integrity all playing essential roles [39,45,89]. Anatomical site is a major determinant of tape strip lipidomic output, as skin lipid abundance and class composition vary markedly across body regions because of sebaceous gland density and epidermis turnover. Therefore, biomarker studies must standardize sites or analyze sites separately to avoid confounding site-specific lipid biology with disease effects [47]. In cholestatic pruritus, scratching behavior and barrier function can further complicate interpretation, making it difficult to distinguish disease-specific lipid changes. The SC lipidome is also influenced by age, sex, and skin hydration [48]. For example, aging is associated with reduced hydration, altered ceramide composition, and barrier decline, whereas sex and hydration status further modulate lipid abundance and class distribution. These variables should therefore be considered carefully in biomarker studies if cases and controls differ by age, sex, or baseline hydration. The optimal design is to match age and sex, measure hydration or transepidermal water loss (TEWL) at collection, and include these as covariates in analyses. Without such control, there is a risk of attributing normal demographic or barrier state variation to disease biology.

Seasonal variation is another important preanalytical confounder in tape stripping lipidomics for cholestatic pruritus because the stratum corneum lipid profile changes with season [93]. In cholestatic pruritus studies, this is a concern since the symptom itself fluctuates with seasonal changes, skin dryness, and stress, which can overlap with seasonal shifts. As a result, a lipid biomarker signal may reflect coldness-associated xerosis or seasonal barrier remodeling rather than cholestasis-specific pruritogen itself. For this reason, it is recommended that studies balance case and control sampling across seasons or treat season as a covariate when interpreting lipidomics data.

Beyond biological variability, technical limitations can also complicate SC lipidomic profiling. Tape-to-tape variability, inconsistent sampling depth, and differences in pressure during tape application can all affect lipid recovery and reproducibility [45,47,81]. Sequential tape strips remove different amounts of stratum corneum, and the amount of cells collected can vary, especially in the top layers that are collected [45]. However, normalization strategies remain incompletely standardized, with previous studies varying in strategies, including protein content, tape weight, lipid abundance, and SC depth [47,48,82]. Contamination from sebaceous lipids, environmental contaminants, or adhesive-derived polymers could also interfere with lipid detection and quantification [47,48,82]. Analytical challenges, including ion suppression, isobaric overlap, incomplete spectral libraries, and uncertainty in lipid annotations, could further complicate identification of low-abundance lipids [83,84,85,86,87,88]. These technical and analytical constraints are particularly relevant for low-abundance lipid species, such as bioactive mediators and pruritogens, where extraction efficiency and ion suppression can limit detection and structural confidence. To address these analytical challenges, potential solutions include standardizing tape type, the number of strips collected, and application pressure; separating sebaceous-rich from sebaceous-poor sites; normalizing to a stable proxy such as protein, total phosphorus, or a validated internal standard; confirming lipid identities by MS/MS rather than accurate mass alone; and using QC pools throughout the analysis.

In addition to biological and technical limitations, challenges in clinical applications also remain. Current skin tape studies are mostly observational, making it difficult to establish the causality between SC lipid alterations and itch pathogenesis. With this in mind, a change in SC lipids should be interpreted as a candidate marker of disease biology, not proof of a causal pruritogenic mechanism. Longitudinal and mechanistic studies (e.g., paired with serum/plasma biomarkers) will be vital in clarifying these relationships. Additionally, SC findings may reflect local skin processes rather than systemic origin for direct deposition of circulating pruritogens. Likewise, more studies are required to prove that a biomarker is reproducible, discriminative, and actionable in real patients. So, for clinical application, the likely use case is stratification or monitoring of CP patients.

Despite these challenges, advances in analytical chemistry, sampling standardization, and lipidomics are likely to improve biological interpretation and subsequent reproducibility. As advances occur, SC lipidomics has the potential to offer insight into the metabolic mechanisms underlying cholestatic pruritus and ultimately support the development of effective therapies.

## 8. Conclusions and Future Directions

In cholestatic liver disease, pruritogens or pro-pruritogenic substances presumed to be produced in the liver are transported through the systemic circulation to the skin. Lysophosphatidic acid (LPA), bile acids, steroids and bilirubin have all been implicated as the cause of the itch that initiates itch signaling pathways in the skin. Consequently, the SC, as the outermost layer of the skin, represents a biologically relevant target tissue for exploring the underlying etiology of pruritus. Although significant work remains before a combined tape stripping and lipidomics platform becomes a first-line, point-of-care test, the approach offers a vital window into the cutaneous molecular landscape of itch. Ultimately, these strategies could transform cholestatic pruritus from a subjective and debilitating symptom into a measurable and treatable condition.

## Data Availability

No new data were created or analyzed in this study. Data sharing is not applicable to this article.
